# Supplementary material for: Phosphoproteomic Landscape of AML Cells Treated with the ATP-Competitive CK2 Inhibitor CX-4945
Source: Cells. 2021 Feb 5;10(2):338. doi: 10.3390/cells10020338 (PMC7915770; doi:10.3390/cells10020338)
Supplement: Supplementary file 1 [file cells-10-00338-s001.zip › Supplementary Figures.docx]

**Supplementary Figures**

**
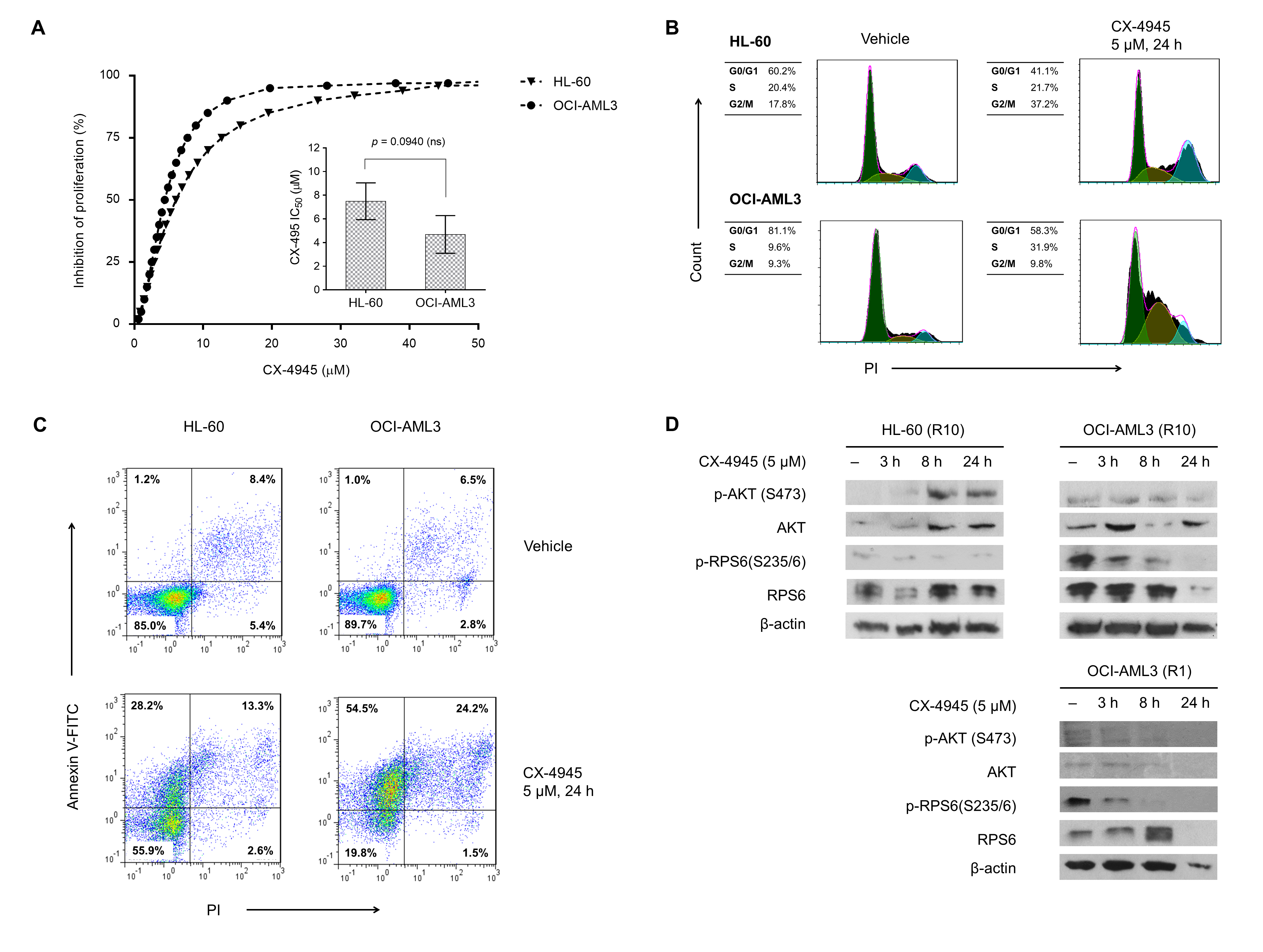
**

**Figure S1.** CK2 inhibitor CX-4945 impairs proliferation and viability of AML cells: (**a**) Proliferation of HL-60 and OCI-AML3 cells was assessed using AlamarBlue assay. Dose-response curves are representative of three independent experiments and IC_50_ values are shown as mean ± SD, n = 3. ns, not significant; (**b**) Analysis of cell cycle distribution was conducted after 24 h of incubation of AML cells with 5 µM CX-4945. The inhibitor induced accumulation of HL-60 cells in G2/M phase, whereas accumulation of OCI-AML3 cells in S phase was observed; (**c**) Viability of HL-60 and OCI-AML3 cells was measured by Annexin V/PI staining. Following 24 h of incubation, CX-4945 induced apoptosis in both AML cell lines; however, the percentage of apoptotic cells was higher in OCI-AML3 treated cells when compared to HL-60 cells; (**d**) Immunodetection of PI3K/AKT signaling pathway mediators in HL-60 and OCI-AML3 cells treated with 5 µM CX-4945. Experiments were conducted in the presence of serum 10 % v/v (R10) or 1 % v/v (R1). To note, we were unable to detect the selected PI3K/AKT mediators in HL-60 cells under R1 conditions.

**
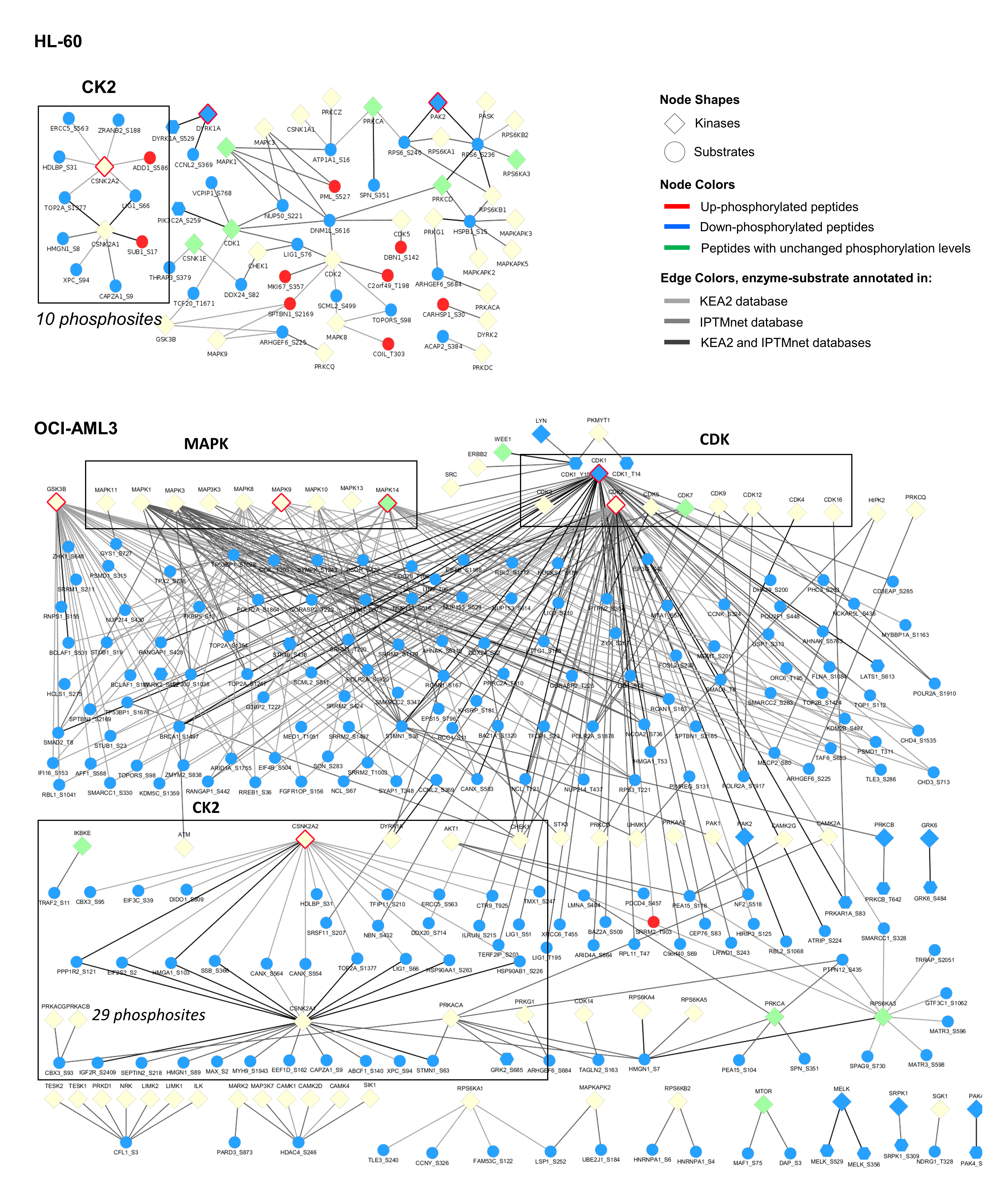
**

**Figure S2.** Enzyme-substrate network of differentially modulated phosphopeptides identified in AML cells using annotations from iPTMnet and KEA2. In squares are indicated the MAPK and CDK families as well as the CK2 substrates differentially modulated in HL-60 and OCI-AML3 cells after CK2 inhibition by CX-4945. Kinases that are significantly associated with the phosphoproteomic profiles, according to KEA2 results, are highlighted with a red border. Network includes all kinases associated to AML cells modulated phosphoproteome, however green and blue nodes correspond to those kinases that were identified in our analysis with either not differentially modulated phosphopeptides and down-phosphorylated peptides, respectively.


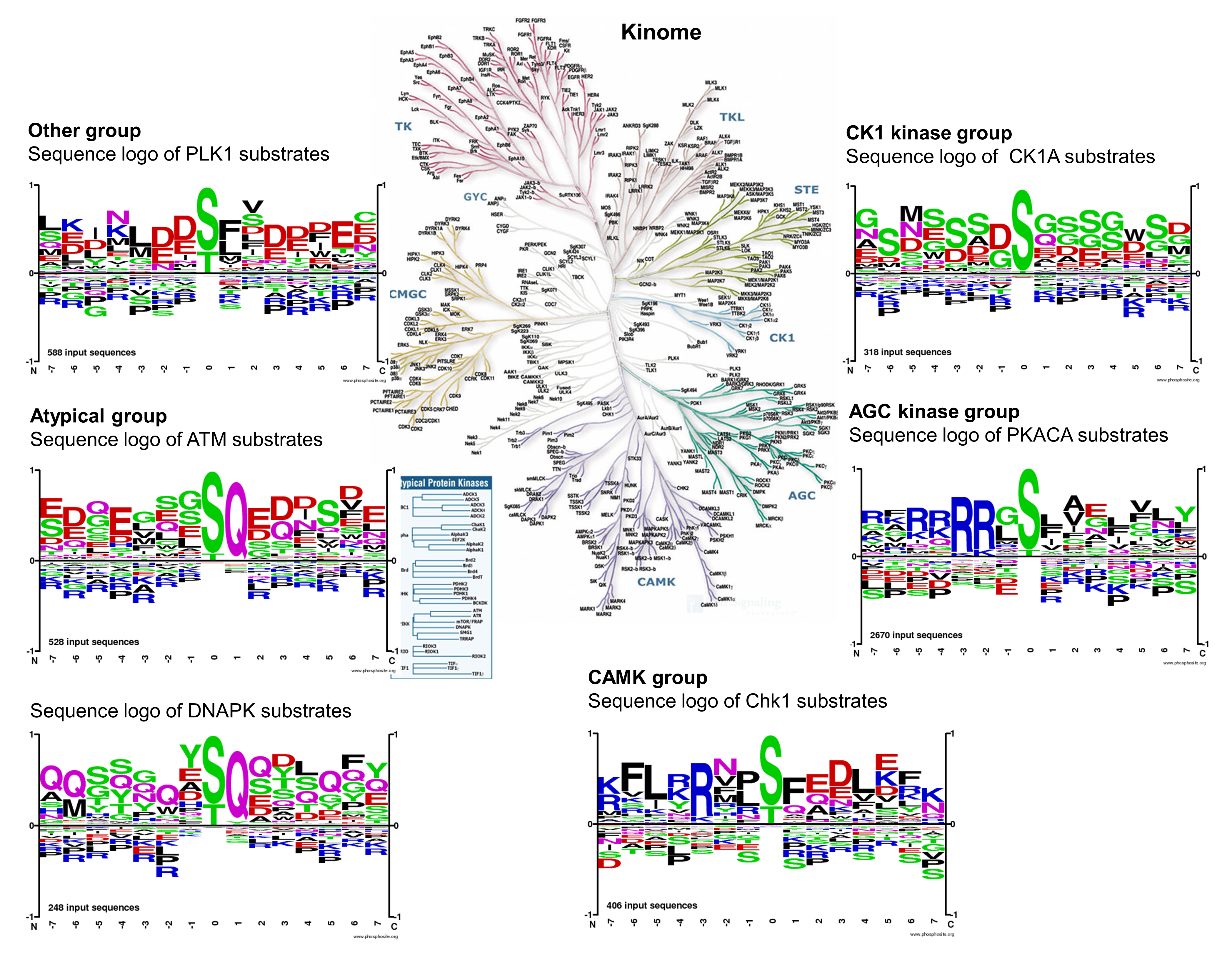


**Figure S3.** Sequence logos of phosphopeptides targeted by protein kinases representing five kinase groups (CAMK, Atypical, CK1, AGC and other) in the human kinome. The analysis was performed using the Motif Analysis tool of PhosphositePlus (PSP) (<http://www.phosphosite.org/>). All the substrates annotated in PSP database for each kinase were used as input sequences.
