# Supplementary material for: Phosphoproteomic Landscape of AML Cells Treated with the ATP-Competitive CK2 Inhibitor CX-4945
Source: Cells. 2021 Feb 5;10(2):338. doi: 10.3390/cells10020338 (PMC7915770; doi:10.3390/cells10020338)
Supplement: Supplementary file 1 [file cells-10-00338-s001.zip › Supplementary Methods.docx]

**Supplementary Methods**

AlamarBlue Assay

The antiproliferative effect of CX-4945 on HL-60 and OCI-AML3 was assessed using AlamarBlue assay (Life Technologies, CA, USA). Briefly, cells were seeded in flat-bottom 96-well plates (2 × 10^5^ cells/mL, 200 µL/well) and 24 h later serial dilutions 1:2 ranging from 50-1.6 µM of CX-4945 (Selleck Chemicals, TX, USA) were added. After 72 h of incubation, AlamarBlue was added at 10% (v/v), and cell suspensions were incubated for 4 h. Finally, fluorescence was measured using a CLARIOstar microplate reader (BMG LABTECH, Ortenberg, Germany) and half-inhibitory concentration (IC_50_) values were estimated using CalcuSyn software (v2.1) (Biosoft, Cambridge, United Kingdom).

Cell Cycle Analysis

For cell cycle analysis HL-60 and OCI-AML3 cells were incubated with 5 µM CX-4945 for 24 h. Following treatment with the inhibitor, cells were collected by centrifugation, washed with PBS and fixed at 4 °C for 30 min with ice-cold 70% ethanol. Cells were treated with DNase-free RNase A (Sigma, MO, USA) and subsequently stained at 37 °C for 20 min with 50 µg/mL PI solution (Sigma, MO, USA). Stained cells were analyzed in Partec CyFlow Space instrument (Sysmex Partec GmbH, Gorlitz, Germany) and FlowJo software (v7.6.1) (BD, Ashland, OR, USA) was used for data analysis and visualization.

Annexin V/PI Staining

Viability of AML cells was measured using FITC Annexin V Apoptosis Detection Kit I (BD Biosciences, CA, USA). Briefly, HL-60 and OCI-AML3 cells were incubated with 5 µM CX-4945 for 24 h. Cells were washed twice with cold PBS and resuspended in binding buffer (1×) at a final concentration of 1 × 10^6^ cells/mL. Subsequently, 5 µL of FITC Annexin V and PI were added and cells suspensions were incubated for 15 min at room temperature in the dark. Flow cytometric analysis of stained cells was performed in abovementioned Partec CyFlow Space instrument and FlowJo Software (v7.6.1) was used for data processing and visualization.

Western Blot

Cells were lysed in RIPA buffer containing protease/phosphatase inhibitor (Thermo Fisher Scientific, MA, USA), and equal amounts of protein were resolved in 12.5% SDS-PAGE [[47](#_ENREF_47)]. Next, proteins were transferred to a nitrocellulose membrane and immunoblotted with the following antibodies according to instructions from the manufacturer: p-AKT (S473), AKT, p-RPS6 (S235/6), RPS6 (Cell Signaling Technology, MA, USA), and β-actin (Sigma, MO, USA). Detection was performed with peroxidase-conjugated anti-rabbit or anti-mouse IgG (Sigma, MO, USA), and signal was developed using SuperSignal West Pico Chemiluminescent Substrate (Thermo Fisher Scientific, MA, USA).
